# Unusual solid submucosal angioleiomyoma of the upper lip: A case report and review of the literature

**DOI:** 10.1097/MD.0000000000046550

**Published:** 2025-12-26

**Authors:** Ruihong Liu, Baolin Jia

**Affiliations:** a Department of Otorhinolaryngology-Head and Neck Surgery, West China Second University Hospital, Sichuan University, Chengdu, China; b Key Laboratory of Birth Defects and Related Diseases of Women and Children (Sichuan University), Ministry of Education, Chengdu, China; c Department of Oral and Maxillofacial Surgery, Suining Central Hospital, Suining, China.

**Keywords:** histopathology, oral soft tissue neoplasms, smooth muscle tumor, solid subtype

## Abstract

**Rationale:**

Angioleiomyoma (ALM) is a rare benign tumor originating from smooth muscle tissue, and its occurrence in the oral cavity is extremely uncommon. Although a few cases involving the upper lip have been reported, most lesions are exophytic or superficially located. Here, we report an unusual solid, submucosal ALM in the upper lip, highlighting its distinctive clinical, imaging, and histopathological features.

**Patient concerns:**

A 43-year-old male presented with a firm, painless swelling in the upper lip that had gradually enlarged over the past 8 months. The lesion measured 4.0 × 2.0 cm, was located in the submucosa, and exhibited no overlying mucosal changes, which made clinical detection challenging.

**Diagnoses, interventions, and outcomes:**

Contrast-enhanced computed tomography demonstrated a well-defined submucosal mass with multiple calcifications. Histopathology classified the tumor as the solid subtype of ALM, showing interlacing bundles of smooth muscle cells surrounding vascular channels, with smooth muscle actin and desmin positivity and S-100 negativity. Complete surgical excision was performed under general anesthesia. The postoperative course was uneventful, and no recurrence was observed during 6 months of follow-up.

**Lessons:**

This case highlights the importance of including ALM in the differential diagnosis of deeply seated upper lip masses. Awareness of its clinical and histopathological characteristics can aid in early diagnosis and appropriate management.

## 1. Introduction

Angioleiomyoma (ALM) is a rare benign tumor characterized histologically by well-differentiated smooth muscle cells surrounding variably sized vascular channels.^[1]^ Although it can occur in various parts of the body, it is most commonly found in the extremities. ALM is extremely uncommon in the oral cavity, accounting for only 0.06% of all oral lesions,^[2]^ and its occurrence in the head and neck region – particularly the upper lip – is exceedingly rare. Most reported cases in this location have been presented as individual case reports.^[3,4]^ The exact pathogenesis of ALM remains unclear, though several etiologic factors have been proposed, including trauma, infection, hormonal changes, genetic predisposition, and vascular malformations.^[5]^ Here, we present a rare case of submucosal ALM of the upper lip in a middle-aged male. This report aims to highlight its clinical and histopathological characteristics and to emphasize the importance of including ALM in the differential diagnosis of deep-seated upper lip masses.

The head and neck region may present with a variety of soft tissue lesions, ranging from benign mucoceles and fibromas to malignant tumors. These lesions can cause complications such as bleeding, infection, functional impairment, or cosmetic deformities. Management strategies depend on lesion type and size and may include conventional surgical excision or minimally invasive approaches, such as diode laser ablation, which have shown favorable clinical outcomes.^[6–8]^

Here, we describe a rare case of submucosal angioleiomyoma in the upper lip of a middle-aged male patient. This case highlights the clinical and diagnostic features of ALM in an uncommon site and aims to raise awareness of its presentation to avoid misdiagnosis and inappropriate management.

## 2. Case reports

A 43-year-old male presented in December 2024 with an 8-month history of a painless swelling in the upper lip. The lesion had gradually increased in size over time without associated pain, numbness, or other discomfort. The patient had no significant medical history and reported no history of trauma or prior local interventions.

**Figure 1.**
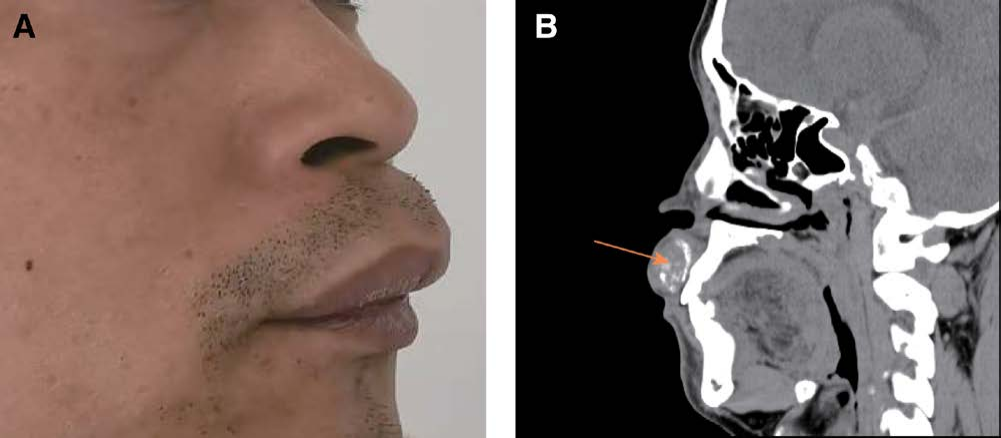
Preoperative clinical photograph and CT findings. (A) Lateral facial view showing swelling of the upper lip. (B) Axial CT image demonstrating a well-defined upper lip lesion with multiple internal calcifications. CT = computed tomography.

Intraoral examination in December 2024 revealed a localized bulge in the upper lip, corresponding to a submucosal mass measuring approximately 4.0 × 2.0 cm on the mucosal side. The overlying mucosa appeared normal. The mass was firm, well-circumscribed, mobile, and non-tender. No loosening or percussion sensitivity of the adjacent teeth was noted (Fig. 1A).

On initial examination, the lesion presented as a firm, well-circumscribed submucosal swelling without tenderness, pulsation, or discoloration of the overlying mucosa. No audible bruit or palpable thrill was detected, suggesting a nonvascular and benign nature. Given its slow growth, firm consistency, and absence of surface changes or pain, the preliminary clinical impression favored a benign soft tissue tumor – such as a fibroma or pleomorphic adenoma – rather than a vascular lesion. Consequently, contrast-enhanced computed tomography (CT) was selected as the initial imaging modality to assess the lesion’s extent and internal architecture. Doppler ultrasonography or magnetic resonance imaging (MRI) was not prioritized at this stage, as no clinical evidence indicated vascular involvement.

Contrast-enhanced CT of the maxillofacial region in December 2024 showed thickening of the upper lip soft tissue with an irregular mass containing multiple calcified foci (Fig. 1B).

**Figure 2.**
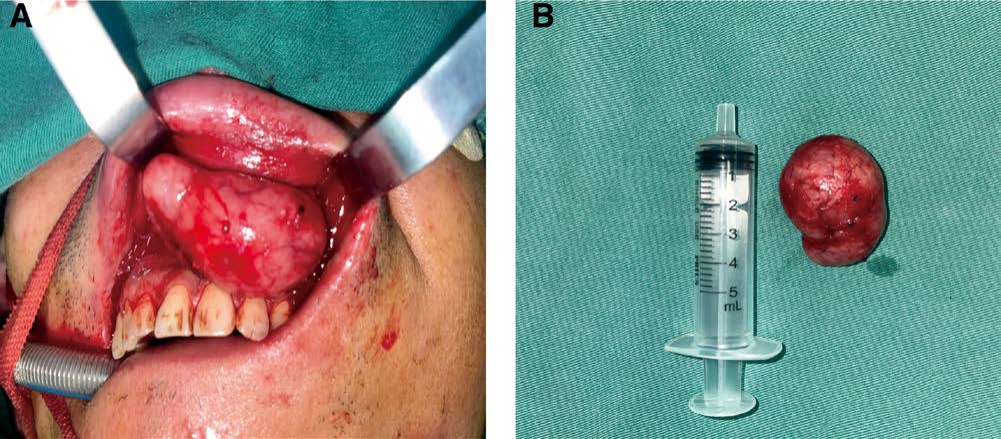
Intraoperative photographs. (A) solid, well-circumscribed tumor with abundant vascular supply and numerous surrounding capillaries. (B) Irregularly oval-shaped lesion with intact capsule, measuring approximately 4.0 × 2.0 cm.

After completing standard preoperative evaluations and excluding surgical contraindications, the patient underwent complete excision of the upper lip mass under general anesthesia in January 2025. An intraoral approach was chosen to preserve lip aesthetics. The incision provided adequate access, but the deep submucosal location and rich vascularity increased surgical difficulty. Intraoperatively, the lesion was solid, well-defined, and highly vascular. Assistant-applied pressure on the labial artery and careful use of bipolar cautery were crucial to control bleeding. Meticulous interrupted suturing and placement of a drain were performed. Postoperatively, local compression dressing using a quadriceps bandage was applied. These measures ensured hemostasis, minimized hematoma formation, and are recommended for similar cases (Fig. 2A and B).

**Figure 3.**
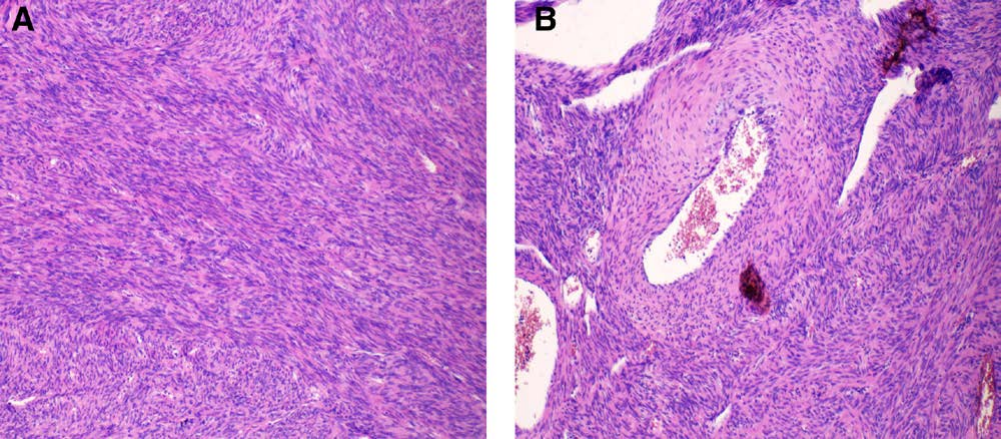
Postoperative HE staining (×100). (A) Tumor composed of interlacing bundles of uniform smooth muscle cells with eosinophilic cytoplasm and minimal atypia. (B) Multiple irregular vascular channels of varying sizes, surrounded by smooth muscle cells merging with the tumor stroma. HE = hematoxylin-eosin.

**Figure 4.**
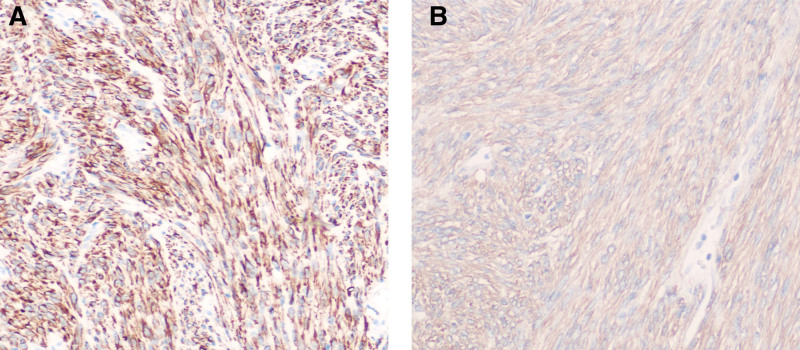
Postoperative immunohistochemical staining (×200). (A) Desmin shows strong and diffuse cytoplasmic positivity in smooth muscle cells. (B) SMA staining shows moderate positivity, with scattered and focally distributed stained cells. SMA = smooth muscle actin.

Histopathological examination of hematoxylin and eosin (H&E)–stained sections revealed interlacing bundles of smooth muscle cells surrounding variably sized vascular spaces with rich vascularity (Fig. 3A and B). No atypical mitotic figures or stromal atypia were observed, supporting the benign nature of the lesion. Immunohistochemical analysis showed diffuse positivity for smooth muscle actin and desmin, while CD34 was positive in vascular endothelium. S-100, SOX-10, and STAT6 were negative. The Ki-67 proliferation index was low (<5%) (Fig. 4A and B).

The final diagnosis was angioleiomyoma of the upper lip. The patient recovered well postoperatively, and no recurrence was observed at the latest follow-up in July 2025, approximately 6 months after surgery, as confirmed by local hospital review and telephone follow-up.

## 3. Discussion

ALM is a benign soft tissue tumor originating from smooth muscle cells surrounding vascular endothelium. Although it predominantly occurs in the extremities, especially the ankle and wrist, the head and neck region – particularly the auricle, lip, and nasal cavity – is the most common site of ALM involvement in this area.^[9,10]^ ALM typically presents as a painless, slow-growing mass, but some lesions may cause pain related to cold or pressure stimuli, possibly due to smooth muscle spasms.^[9]^ The tumor’s higher incidence in females and its association with menstrual cycles and pregnancy suggest hormonal influences in its pathogenesis.^[11,12]^ However, most cases reported in the oral cavity are relatively small and superficial, while submucosal forms are uncommon and may be easily overlooked during clinical inspection. In the head and neck region, the gender distribution of angioleiomyoma (ALM) shows some variation. Liu et al^[10]^ reported a slightly higher proportion of male patients (male:female = 1.625:1), and Bubola et al^[13]^ also found a male predominance (male:female = 3.2:1). These differences may be related to sample size, geographic region, ethnic background, and study methodology. Therefore, the gender distribution of ALM remains inconclusive, and further multicenter studies with larger sample sizes are needed to clarify this issue.

Imaging studies such as CT and MRI are valuable for preoperative assessment, providing information on tumor location, morphology, size, vascularity, and relation to adjacent structures. ALMs usually appear as well-defined, round or oval masses in the deep dermis or subcutaneous tissue, often containing calcifications. MRI features include iso- to slightly hyperintense signals on T1-weighted images and heterogeneous high signals on T2-weighted images, with peripheral capsules and heterogeneous enhancement after contrast administration, reflecting their rich vascularity.^[14]^ The “dark reticular sign” on T2-weighted imaging may serve as a specific radiologic feature of ALM.^[15]^ In our case, the lesion demonstrated multiple calcifications and well-defined margins on CT, consistent with previous imaging descriptions, further supporting the diagnosis.

Histologically, ALM is classified into solid, venous, and cavernous types. The solid type, most common in females, consists of tightly packed smooth muscle bundles surrounding collapsed vascular channels. The venous type, more frequent in males, features thick-walled veins with smooth muscle cell layers, while the rare cavernous type contains dilated vascular spaces with sparse smooth muscle cells.^[16,17]^ In the oral and maxillofacial region, the venous subtype is more commonly reported. However, our case was classified as the solid type, which is relatively uncommon in this anatomical location. Immunohistochemical staining typically shows positivity for smooth muscle actin, calponin, desmin, and endothelial markers CD31 and CD34, with negativity for HMB-45 and S-100.^[17,18]^ Our case’s immunoprofile aligns with these characteristics. This finding broadens the pathological spectrum of lip ALMs, suggesting that solid type lesions, although rare, can occur in submucosal regions of the upper lip.

A review of the literature reveals that angioleiomyoma involving the lip is rare,^[4,17,19–22]^ with only a few well-documented cases reported to date (Table 1). Most lesions were small (approximately 1 cm), well-circumscribed, and presented as painless, exophytic, or mobile nodules. However, the clinical presentation may be influenced more by the anatomical site of involvement, such as tissue thickness and submucosal depth, rather than the tumor subtype itself. Surgical excision was the treatment of choice in all cases, and no recurrence was reported during follow-up (Table 1). Our case differs from these reports by presenting a considerably larger (4.0 × 2.0 cm) and deeply located submucosal mass, with abundant intraoperative vascularity and calcification, which complicated both diagnosis and excision. By documenting these distinctive features, this report contributes to the understanding of the clinical spectrum of oral ALM, emphasizing that submucosal ALM should be considered in the differential diagnosis of deep-seated upper lip swellings that lack surface mucosal changes.

**Table 1:** Summary of reported cases of lip angioleiomyoma in the literature.

Author	Age/Sex	Site	Size	Clinical appearance	Treatment	Outcome
Gueiros et al^[19]^	53–66/Male	Upper lip (2 cases); Lower lip(1 cases)	1.0 cm	Exophytic or pedunculated, painless lesions	Surgical excision	No recurrence
Yazan Hassona et al^[20]^	52/Female	Upper lip	1.0 cm	Submucosal, painless nodule	Excision	No recurrence
Luiz Arthur Barbosa da Silva et al^[21]^	44/Male	Upper lip	1.0 cm	Submucosal, well-circumscribed nodule	Complete excision with layered closure	No recurrence
Matiakis et al^[17]^	51/Female	Upper lip	0.8 cm	Mobile, painless nodule	Surgical excision	No recurrence
Berislav Perić et al^[22]^	36/Male	Upper lip	0.5 cm	Slow-growing, mobile mass	Excision	No recurrence
Payal Dilipkumar Mehta et al^[4]^	57/Male	Lower lip	1.0 cm	Exophytic, nodular, reddish-purple	Excision	No recurrence

While imaging provides valuable anatomical information, histopathological and immunohistochemical confirmation remains essential for diagnosis. The main differential diagnoses include hemangioma, myopericytoma, angiomyolipoma, neural tumors, leiomyosarcoma, and fibrous lesions, which can be distinguished by their distinct histological and immunophenotypic features.^[15,16]^ Specifically, hemangiomas are usually compressible, bluish lesions that blanch on pressure and are more common in younger patients, differentiating them clinically from ALM. Myopericytomas demonstrate concentric perivascular proliferation of oval to spindle-shaped cells, which differs from the interlacing smooth muscle bundles seen in ALM. Leiomyosarcomas may show cytologic atypia, necrosis, and higher mitotic activity, all absent in our case. Angiomyolipomas contain mature adipose tissue in addition to smooth muscle and thick-walled vessels, which was not observed. Neural tumors, such as schwannomas, typically express S-100 protein, which was negative here. Complete surgical excision with capsule preservation is the treatment of choice. Although recurrence and malignant transformation are rare,^[17]^ preoperative planning should anticipate intraoperative bleeding,^[18,19]^ and postoperative follow-up for at least 1 year is recommended.^[10]^ In our patient, complete excision led to satisfactory healing with no recurrence after 6 months.

Although trauma, hormonal influences, and genetic predisposition have been proposed as potential etiological factors for angioleiomyoma, no history of local trauma, systemic disease, or medication use was reported in our patient, and no relevant family history was identified. This suggests that, at least in this case, the tumor may have developed sporadically. The absence of identifiable risk factors underscores the multifactorial and incompletely understood nature of angioleiomyoma pathogenesis. Future studies are warranted to clarify the mechanisms underlying tumor development, particularly in the oral and maxillofacial region.

The patient reported satisfaction with the surgical outcome and cosmetic result. He expressed relief that the swelling and discomfort resolved completely and that no recurrence was observed at follow-up. He also appreciated the clear explanation of the diagnosis and treatment process provided by the medical team.

In retrospect, the initial clinical impression in our case was that of a benign soft tissue tumor, and vascular lesions such as angioleiomyoma were not prioritized in the differential diagnosis. Consequently, Doppler or MRI evaluation was not undertaken before surgery. This case underscores the importance of assessing for vascularity, including the presence of bruit or pulsation, in all soft tissue masses to avoid potential intraoperative bleeding risks. If we were to manage a similar case again, early consideration of vascular lesions and adjunctive Doppler or MRI imaging would be incorporated into the diagnostic workup.

In conclusion, this report adds to the growing body of literature by reporting one of the largest submucosal ALMs of the upper lip described to date. The case highlights not only the importance of considering this rare tumor in differential diagnoses but also the surgical implications of its rich vascularity and deep location. Recognition of this atypical presentation may prevent misdiagnosis and guide appropriate surgical planning. Careful preoperative planning and complete excision remain critical to ensuring both diagnostic clarity and long-term prognosis.

## Author contributions

**Writing – original draft:** Ruihong Liu.

**Writing – review & editing:** Baolin Jia.
